# Microglial inhibition alleviates alpha-synuclein propagation and neurodegeneration in Parkinson’s disease mouse model

**DOI:** 10.1038/s41531-024-00640-2

**Published:** 2024-02-02

**Authors:** Thuy Thi Lai, Young Eun Kim, Linh Thi Nhat Nguyen, Tinh Thi Nguyen, In Hee Kwak, Franziska Richter, Yun Joong Kim, Hyeo-il Ma

**Affiliations:** 1 Hallym Neurological Institute, Hallym University, Anyang, Gyeonggi 14068 South Korea; 2 Department of Pharmacology, Toxicology and Pharmacy, University of Veterinary Medicine, 30559 Hannover, Germany; 3 Center for Systems Neuroscience Hannover, Hannover, Germany; 4 Department of Neurology, Hallym University Sacred Heart Hospital, Hallym University, Anyang, Gyeonggi 14068 South Korea; 5 Department of Neurology, Yongin Severance Hospital, Yonsei University College of Medicine, Yongin, Gyeonggi South Korea

**Keywords:** Parkinson's disease, Neurodegeneration

## Abstract

The accumulation of alpha-synuclein (αSyn) is widely recognized as the main pathological process in Parkinson’s disease (PD). Additionally, neuroinflammation is considered to be one of the contributing mechanisms in the development of PD. In light of this, it is hypothesized that the reactive microglia exacerbate the propagation of αSyn and neurodegeneration, while the inhibition of microglial activity may mitigate these effects. To test this hypothesis, αSyn preformed fibrils (PFF)-injected PD mouse model was employed. Co-injection of lipopolysaccharide (LPS) and PFF was performed to investigate if microglial reactivity intensified αSyn propagation and neurodegeneration. Additionally, oral administration of PLX5622, a microglial inhibitor that targets the colony-stimulating factor 1 receptor, was given for two weeks before and after PFF injection each to explore if microglial inhibition could prevent or reduce αSyn pathology. Intrastriatal co-injection of LPS and PFF resulted in increased microglial reactivity, αSyn accumulation, and neurodegeneration compared to PFF injection alone. However, treatment with PLX5622 significantly suppressed microglial reactivity, reduced αSyn pathology, and alleviated dopaminergic neuron degeneration in the PD mouse model injected with PFF. Based on these findings, it is evident that microglial reactivity plays a crucial role in the progression of αSyn pathology and neurodegeneration in PD. Furthermore, the results suggest that microglial inhibition may hold promise as a therapeutic strategy to delay the progression of αSyn pathology in PD.

## Introduction

Parkinson’s disease (PD) is a debilitating neurodegenerative disorder characterized by the accumulation of alpha-synuclein (αSyn) within neurons and the loss of neuronal cells^1^. The precise mechanisms through which these proteins aggregate and surpass pathological thresholds, leading to neurodegeneration, remain unclear. However, several contributing factors have been identified, including neuroinflammation, dysfunctional protein clearance, and mitochondrial dysfunction^1^.

Among these mechanisms, neuroinflammation has emerged as a critical component of the pathogenesis of PD^2^. This association was first observed in the autopsied brain of PD patients and supported by data from cerebrospinal fluid or peripheral blood, and genetic studies^2–4^. Notably, microglial reaction was consistently observed in these studies, in contrast to reports of astrocytic reaction^2^.

Furthermore, animal studies have revealed that microglial reaction, specifically, is preferentially increased in αSyn preformed fibril (PFF) injected PD mouse model, compared to astrocytic reaction^5^. Additionally, observation of embryonic dopaminergic neuron transplantation in PD revealed that microglial reactivity occurred in graft deposits well before the onset of αSyn accumulation in implanted dopamine neurons^6^.

These findings suggest that microglial reactivity may contribute to the progression of synucleinopathy, prompting our investigation into whether microglial reactivity exacerbates αSyn propagation and neurodegeneration, and whether inhibition of microglial reactivity could serve to mitigate these pathological processes.

## Results

**Fig. 1:**
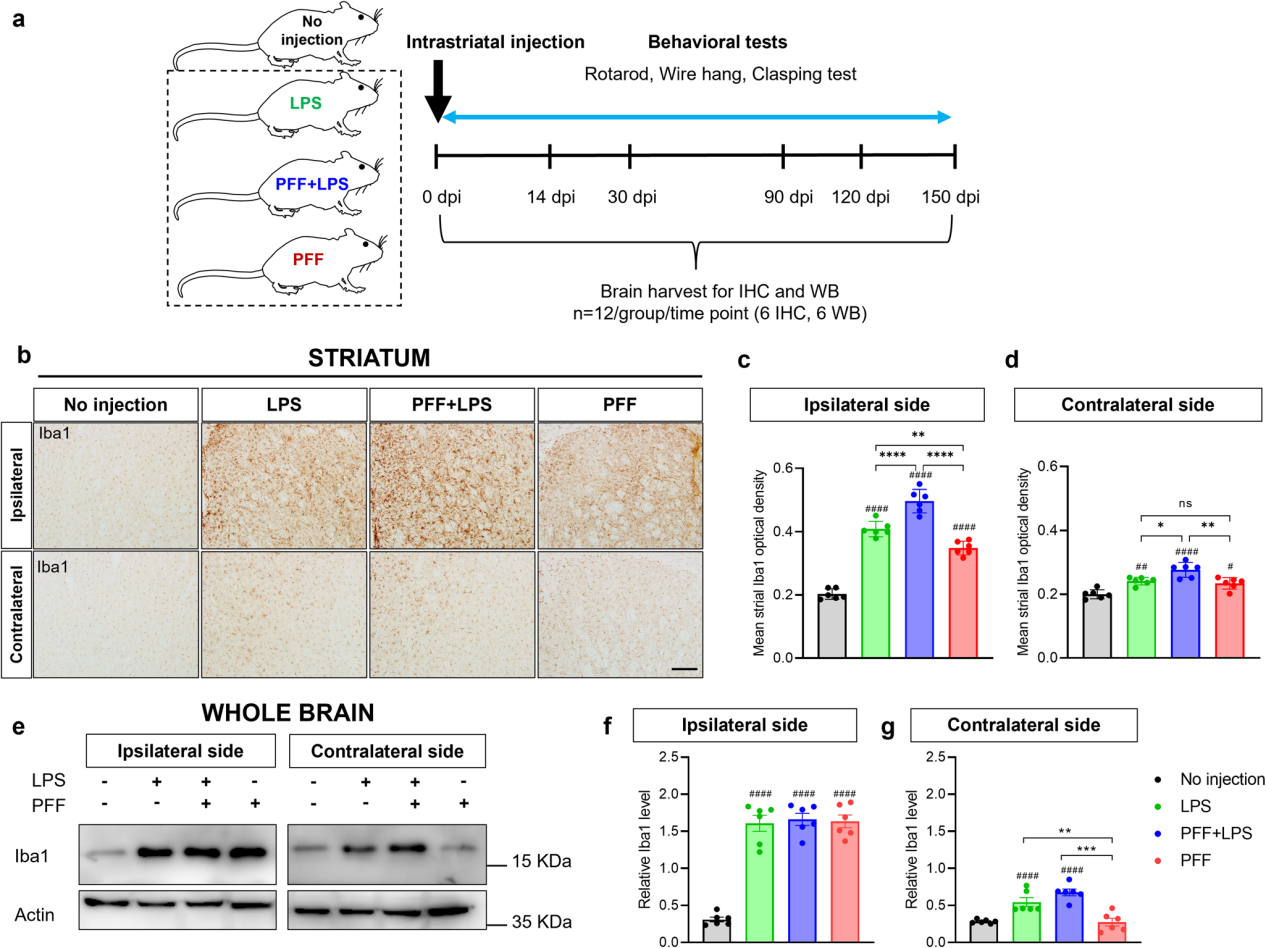
LPS injection exacerbates microglial reactivity in the PFF-injected PD mouse model. **a** Overview of the experimental scheme. **b**–**d** Microglial reactivity in bilateral striatum at 14 days post-injection. **b** Representative immunostaining for Iba1 in bilateral striatum, Scale bar = 200 μm. **c**, **d** Quantification of Iba1 using densitometric analysis. **e**–**g** Microglial reactivity in whole brain homogenates at 14 days post-injection. **e** Representative western blot image of Iba1. **f**, **g** Quantification of Iba1 using western blot. Error bars represent mean ± SEM. Statistical analyses were performed using one-way ANOVA followed by a post-hoc test for multiple comparisons, whereas (#) denotes a significant difference compared with no injection control, and (*) denotes a significant difference compared to the other animal groups. Detailed statistics are provided in Supplementary Table 1. Abbreviations: IHC, immunohistochemistry; WB, western blot.

In pursuit of investigating the progression of synucleinopathy in PD, we generated mouse models by injecting PFF, and inducing heightened microglial reactivity with the addition of pro-inflammatory bacterial lippopolysaccharide (LPS). We sacrificed three groups of mice at different time points (14, 30, 90, 120, and 150 dpi) – those injected with LPS only, those injected with PFF with LPS, and those injected with PFF only, while a comparison group without any injection was also sacrificed (Fig. 1a).

### LPS injection exacerbates microglial reactions

In mouse models of PD, microglial reactivity is aggravated by LPS injection, as observed in the striatum at 14 dpi (Fig. 1b–d). Microglial reactivity was also present in mice injected with PFF only, but the injection of PFF with LPS resulted in a higher level of microglial reaction compared to PFF-only injection (Fig. 1b–d). This observation was further supported by western blot analysis using whole brain lysates (Fig. 1e–g). Furthermore, western blot analysis detected a significant enhancement of astrocytic reaction by LPS injection (Supplementary Fig. 1). Taken together, these findings suggest the LPS injection strongly induces additional inflammation, including microglial reactivity, in the PD mouse model.

### LPS injection accelerates the αSyn accumulation in the striatum and substantia nigra

**Fig. 2:**
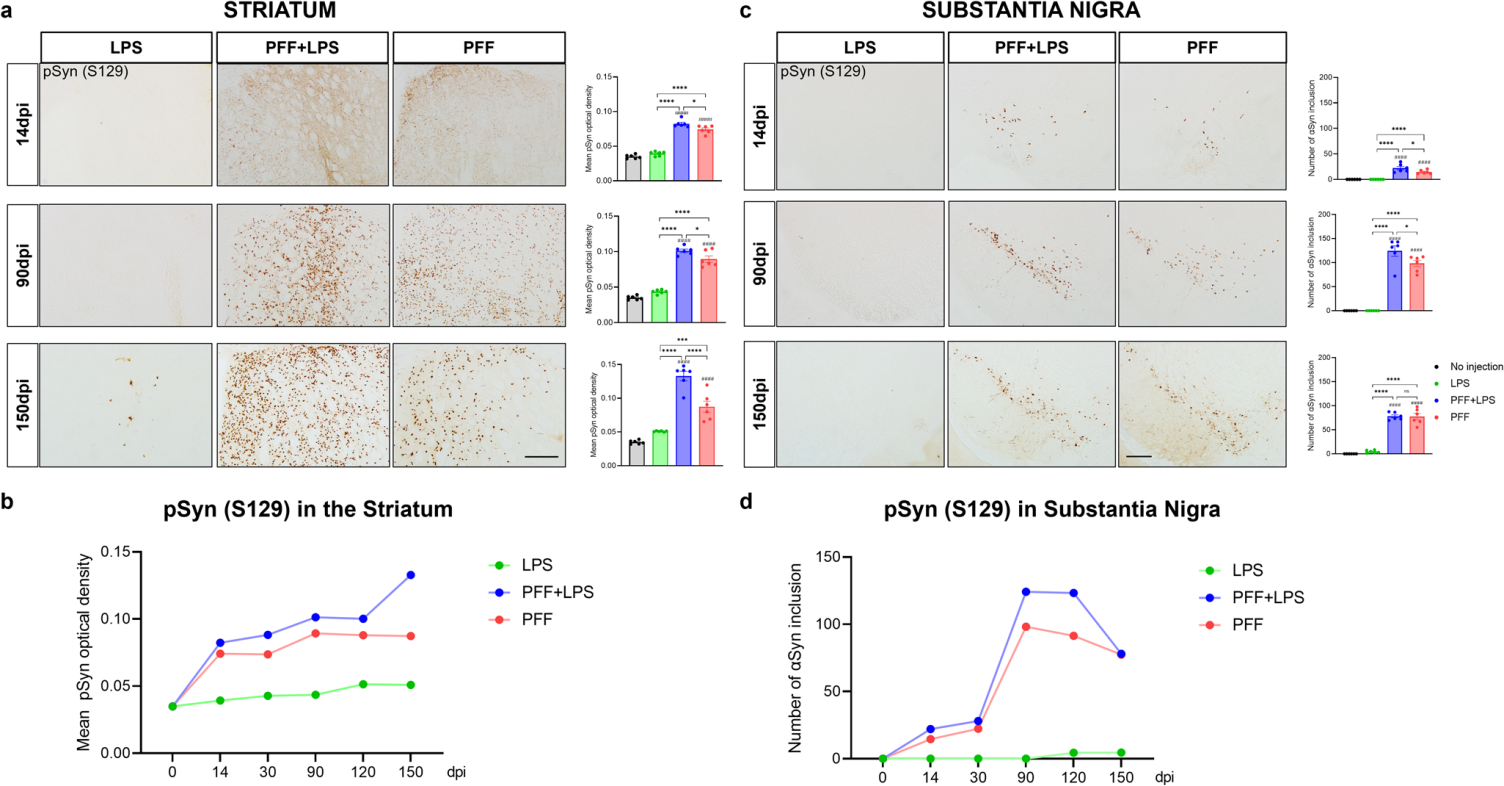
LPS injection accelerates αSyn accumulation in the striatum and substantia nigra. **a** Representative immunostaining for pSyn in the ipsilateral striatum and its quantification of pSyn using densitometry. **b** Temporal change of pSyn density in the ipsilateral striatum. **c** Representative immunostaining for pSyn in the ipsilateral SN and its quantification of pSyn using densitometry. **d** Temporal change of pSyn density in the ipsilateral SN. Scale bar = 200 μm. Error bars represent mean ± SEM. Statistical analyses were performed using one-way ANOVA followed by a post-hoc-test for multiple comparisons, whereas (#) denotes a significant difference compared with no injection control, and (*) denotes a significant difference compared to the other animal groups. Detailed statistics are provided in Supplementary Table 1.

To further investigate the impact of LPS-induced microglial reactivity on αSyn accumulation in a mouse model of PD, we examined brain section of mice injected with either PFF with LPS or PFF only. We observed widespread pSyn-immunopositive signals in various brain regions from 14 dpi to 150 dpi (Supplementary Fig. 2). In particular, pSyn immunoreactivity in the striatum significantly increased in mice injected with PFF with LPS compared to those injected with PFF only, from 14 dpi to 150 dpi (Supplementary Fig. 2 and Fig. 2a, b).

Figure 2b showed a trend of increasing pSyn accumulation in the ipsilateral striatum over time in all three mice groups. Specifically, pSyn immunoreactivity continuously increased in PFF with LPS mice until 150 dpi, while the pSyn immunoreactivity in the PFF-only group plateaued at around 90 days (Fig. 2a, b and Supplementary Fig. 3).

In the substantia nigra (SN), the number of αSyn inclusions was calculated in all three groups (Fig. 2c, d and Supplementary Fig. 3). Our analyses revealed that co-injection of PFF and LPS resulted in significantly higher αSyn inclusion compared to the PFF-only group in the SN, although this difference was not significant at 150 dpi.

Interestingly, sparse pSyn-positive signals were observed in the SN as well as the striatum of mice receiving LPS only at 120 dpi and 150 dpi (Fig. 2a, c and Supplementary Fig. 4a, b). There were no pSyn-positive signals in the no injection group as expected (Supplementary Fig. 3c).

### LPS injection accelerated degeneration of dopaminergic neurons in the substantia nigra

**Fig. 3:**
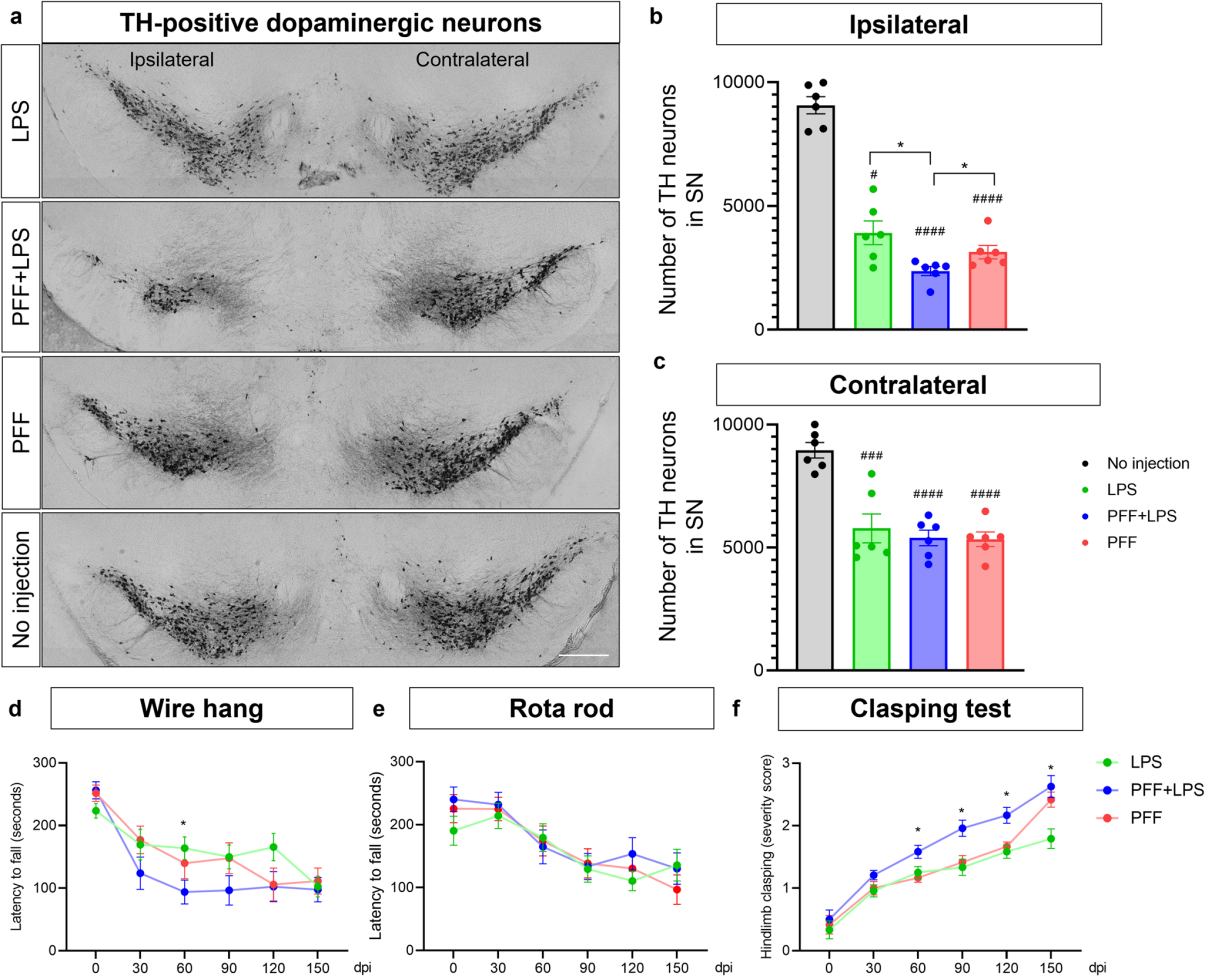
LPS injection accelerates degeneration of dopaminergic neurons in the substantia nigra. **a** Representative image of SN dopaminergic (TH-positive) neurons at 150 dpi. Scale bar = 500 μm. **b**, **c** Stereology count of TH-positive cells in ipsilateral (**b**) and contralateral (**c**) SN compacta. Error bars represent mean ± SEM. Statistical analyses were performed using one-way ANOVA followed by a post-hoc-test for multiple comparisons. **d**–**f** Behavioral assessment at each time point using the wire hang test (**d**), rotarod test (**e**), and hindlimb clasping test (**f**) (*n* = 12 per group). Statistical analyses were performed by using two-way repeated ANOVA followed by a post-hoc-test for multiple comparisons. (*) denotes a significant difference between three different animal models. (#) denotes a significant difference compared to the mice without injection. Detailed statistics are provided in Supplementary Table 1.

To access the loss of dopaminergic neurons in three different groups, we utilized unbiased stereology to estimate the number of tyrosine hydroxylase (TH)-positive cells in the coronal section spanning the SN. All three groups exhibited a significant reduction in dopaminergic neurons in the bilateral SN compared to control mice without any injection at 150 dpi (Fig. 3a–c). At 90 dpi, we estimated a loss of 13.5%, 39.1%, and 32% of dopaminergic neurons in ipsilateral SN of mice injected with LPS-only, PFF with LPS, and PFF-only (Supplementary Fig. 4c, d). At 150 dpi, there was an approximate loss of 56.8%, 73.8%, and 65.4% of dopaminergic neurons in the ipsilateral SN of LPS only, PFF with LPS, and PFF only groups, respectively. Notably, the loss of dopaminergic neurons was significantly greater in the PFF with LPS group compared to the PFF or LPS only groups at 150 dpi, although no significant difference in dopaminergic neurons in the contralateral striatum was detected between the three animal groups (Fig. 3b, c).

We also assessed the development of motor deficits in the three groups. Our finding revealed that animals in all groups exhibited a disease-related decline in motor performance over time in the rotarod and wire hang tests, although there was no significant difference between the PFF with LPS group and the PFF only group in these tests (Fig. 3d, e and Supplementary Table 1). Notably, in the hindlimb clasping test, mice injected with both PFF and LPS exhibited a significantly more severe phenotype compared to the other groups from 60 dpi, as demonstrated in Fig. 3f and Supplementary Table 1.

### Inhibition of microglial reactivity attenuates αSyn accumulation and neurodegeneration in PFF-injected PD mouse model

**Fig. 4:**
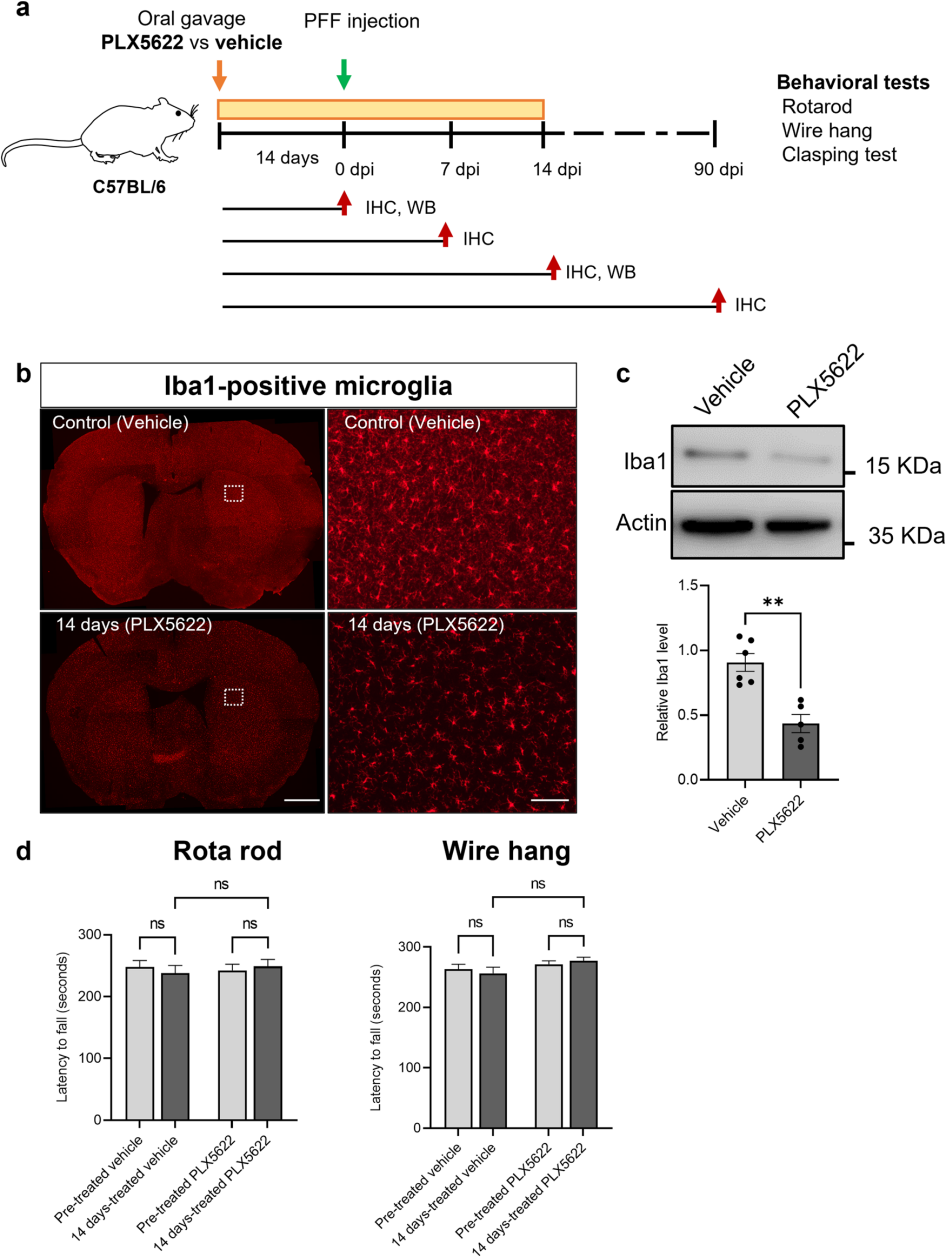
CSF1R inhibitor significantly reduces Iba1-positive microglia and characterization of CSF1R inhibitor treatment in mice. **a** Overview of the experimental scheme. **b** Representative image of the inhibition microglial reaction by PLX5622 treatment at 14 days in the brain. Scale bar = 1 mm and 100 µm. **c** Quantification of Iba1 positivity using western blot. **d** Behavior assessment before and after PLX5622 treatment. Error bars represent mean ± SEM. (*) denotes a significant difference between groups, while (ns) denotes no significant difference between groups. Detailed statistics are provided in Supplementary Table 1.

In the second phase of our study, we investigated the effects of inhibition of microglial reactivity using the colony-stimulating factor-1 receptor (CSF1R) inhibitor (PLX5622) on αSyn accumulation and neurodegeneration in a mouse model of PD induced by PFF injection (Fig. 4a).

### CSF1R inhibitor significantly reduces Iba1-positive microglia

To assess the impact of CSF1R inhibitor on microglial reaction, we treated mice with PLX5622 or vehicles as described in the methods section. After 2 weeks of treatment, we observed a significant reduction in Iba1-positive microglia in the brain of mice treated PLX5622 compared to those treated with vehicle, as demonstrated in Fig. 4b. Furthermore, western blot analysis revealed a significant decrease of 47.95% in Iba1-positive microglial reaction in the whole brain lysate of PLX5622-treated mice, as shown in Fig. 4c. Importantly, we did not observe any significant differences in motor performance between PLX5622-treated and vehicle-treated groups, as depicted in Fig. 4d. These findings confirm that a two-week oral administration of PLX5622 successfully achieves partial microglial depletion, without causing any functional deficits.

Additionally, we confirmed that extended administration of PLX5622 resulted in a partial depletion of microglia even after PFF injection. Mice brains sacrificed at 7 and 14 days after PFF injection showed decreased microglial reactivity in PLX5622-treated mice compared to vehicle-treated mice, while PBS-injected mice treated with PLX5622 also reduced microglial reaction (Supplementary Fig. 5). Furthermore, an observable tendency of diminished microglial reaction was observed in the ipsilateral areas of the striatum, amygdala and SN in mice treated with PLX5622 in comparison to those treated with the vehicle at 90 dpi (Supplementary Fig. 6).

### CSF1R inhibitor decreases αSyn accumulation and neurodegeneration in PD mouse model

**Fig. 5:**
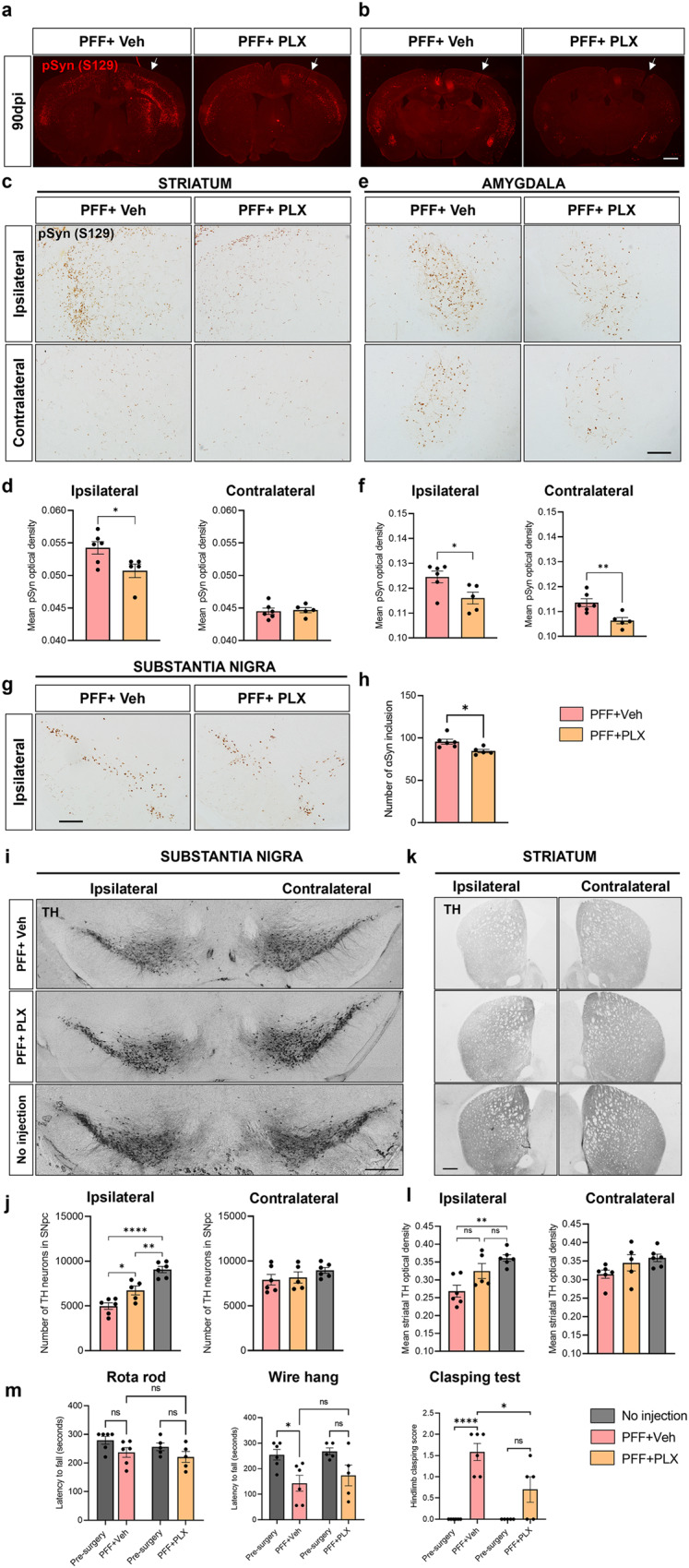
CSF1R inhibitor reduces αSyn accumulation and neurodegeneration in PD mouse model. **a**–**e** Representative immunostaining for pSyn in the striatum (**a**, **c**), amygdala (**b**, **e**) and substantia nigra (**g**) at 90 dpi. Scale bar = 1 mm and 200 µm. The white arrow signifies the ipsilateral side where PFF injection was administered. **d**, **f**, **h** Quantification of pSyn in the striatum (**d**), amygdala (**f**) and substantia nigra (**h**). Statistical analyses were performed using the Mann-Whitney test. **i**–**k** Representative immunostaining for TH-positive neurons in the SN (**i**) and striatum (**k**) at 90 dpi. Scale bar = 500 µm. **j** Stereology count of TH-positive cells in SN. **l** Quantification of TH-positive density in the striatum. **m** Behavioral assessment using rotarod, wire hang, and hindlimb clasping test. Statistical analyses were performed using one-way ANOVA followed by a post-hoc-test for multiple comparisons, where (*) denotes significance and (ns) denotes the non-significant difference between treatments. Error bars represent mean ± SEM. Detailed statistics are provided in Supplementary Table 1.

We examined the effects of PLX5622 on αSyn accumulation and neurodegeneration in a mouse model of PD induced by PFF injection. At 7 dpi and 14 dpi after PFF injection, administration of PLX5622 resulted in a reduction of αSyn accumulation in various brain regions (Supplementary Fig. 7a). At 14 dpi, we observed a decreasing tendency in the soluble and insoluble αSyn contents of the whole brain in PLX5622-treated mice compared to vehicle-treated mice, although the differences did not reach statistical significance (Supplementary Fig. 7b–e). At the long-term point of 90 dpi, we observed a significant reduction in αSyn accumulation in the ipsilateral striatum, bilateral amygdala, and ipsilateral SN of PLX5622-treated mice compared to the vehicle-treated mice (Fig. 5a–h).

Furthermore, we compared the degeneration of dopaminergic neurons between the two groups at 90 dpi. We found a significant decrease in the degeneration of dopaminergic neurons in the ipsilateral side of the SN in PLX5622-treated mice compared to vehicle-treated mice, although both groups showed significant loss of dopaminergic neurons compared to healthy mice (Fig. 5i, j). We also observed an improvement in the attenuation of TH-positive optical density in the striatum of mice receiving PLX5622, as depicted in Fig. 5k, l. Additionally, PLX5622 treatment resulted in a decrease in motor deficit, as measured by the hindlimb clasping test (Fig. 5m).

## Discussion

In this study, we aimed to investigate the effect of microglial reactivity on αSyn accumulation and neurodegeneration in a mouse model of PD. We observed that microglia reactivity exacerbates the propagation of αSyn and neurodegeneration, while inhibition of microglial activity mitigates these effects. A study on a PD mouse model showed that co-injection of LPS and αSyn PFF resulted in greater microglial reaction, αSyn accumulation, and neurodegeneration than PFF injection alone. However, oral gavage treatment with PLX5622, a microglial inhibitor, significantly suppressed microglial reaction, reduced αSyn pathology, and alleviated degeneration of dopaminergic neurons in the PFF-injected PD mouse model.

Our study found a strong correlation between inflammation, particularly microglial reaction, and the propagation of αSyn in a PD mouse model. Autopsy data from humans spanning several decades consistently reported the presence of microglial reaction in postmortem PD brain^7–9^. Additionally, in vivo evidence using PET imaging has supported the presence of microglial reaction in the human PD brain^10–13^. Olanow et al. recently presented autopsy data from PD patients who had received fetal nigral cell transplantation, demonstrating the presence of microglia around the transplanted cells from the start of grafting and even before αSyn accumulation occurred within the transplanted cells^6^. These studies provide support for the idea that microglial reactivity may precede αSyn propagation. Consistent with this evidence, our study demonstrated that microglial reactivity is not merely a side effect of PFF injection or coincidental with propagation, but rather a contributing factor in the deterioration of synucleinopathy.

Microglia, the resident immune cells of the brain, are essential for maintaining brain homeostasis. Previous references suggest that microglia may have diverse roles in the propagation of αSyn. In a quiescent state, microglia can internalize and degrade αSyn through phagocytosis^14^. However, in certain circumstances such as αSyn overexpression, hyperstimulated microglia, which activated Toll-like receptor 2 (TLR2) in response to αSyn, can also contribute to the spread of αSyn pathology by releasing pro-inflammatory cytokines and other molecules that induce oxidative stress and neuronal death^15^. Meanwhile, microglia can modulate αSyn transfer or accumulation in the brain by regulating the activity of the immune cells and producing neurotrophic factors that promote the survival of neurons. As a result, the role of microglia in synucleinopathy is complex, with both protective and harmful effects on αSyn pathology and neurodegeneration.

A recent review has shown that microglia is a diverse set of cells that have the ability to screen the environment and start to differentiate into multiple subtypes in specific responses^16^. It is plausible that different subtypes of microglia may be associated with the diverse role they play in the context of synucleinopathy. We found that microglia are responsive to either LPS or PFF by increasing the density of Iba1-positive microglia. In the presence of both LPS and PFF, microglial density increased remarkably compared to LPS or PFF only. However, the specific subtype of microglia that is implicated in αSyn propagation and neurodegeneration was not identified in our current study. The findings of this study indicate that co-injection of LPS and PFF-induced microglia reactivity contributes to the worsening of synucleinopathy. However, partially suppressing microglia reactivity with a CSF1R inhibitor can alleviate this effect, which indicates microglia reactivity does contribute to PFF-caused pathology in mouse models. Nevertheless, it is crucial to further investigate the molecular subtype or phenotype of microglia associated with αSyn propagation or neurodegeneration, as the beneficial or modifiable role of microglia in its states cannot be disregarded in the context of synucleinopathy.

Even in the absence of pulsed αSyn PFF injection or genetic αSyn overexpression, prolonged microglial reaction or inflammation can trigger synucleinopathy. Our study found that LPS-only injection induced αSyn accumulation in the long term (120 and 150 dpi), consistent with previous findings^17,18^. Niu et al. reported that chronic intranasal LPS treatment induced αSyn aggregation and phosphorylated αSyn in the olfactory bulb, striatum, and SN after 6 weeks^17^. Similarly, Zhao et al. also observed a prominent increase in the immunoreactivity of pSyn (S129) in the SN and hippocampus of wild-type mice 10 months after a single intraperitoneal injection of LPS^18^. These findings highlight that inflammation alone, without PFF injection or genetic modification, can induce αSyn accumulation, underscoring the importance of inflammatory mechanisms in idiopathic PD. It is worth noting that while most cases of PD are idiopathic, with unknown causes, a small portion of PD cases is associated with specific genetic mutations. Therefore, animal models utilizing PFF injection or genetic modification may not fully represent typical PD, further emphasizing the significance of inflammatory processes in idiopathic PD.

To explore the contribution of microglia in αSyn propagation, we administered PLX5622, a colony-stimulating factor 1 receptor inhibitor that suppresses microglia before inducing PD mice by PFF injection in this study. We confirmed that microglial reactivity is suppressed effectively without causing any abnormal motor functions through the administration of PLX5622 orally for duration of two weeks. Our results showed that PLX5622 treatment significantly decreased microglial reactivity and αSyn pathology following PFF injection. Furthermore, microglial inhibition by PLX5622 treatment protected dopaminergic neurons from degeneration in the PFF-injected mouse model. In addition, PFF-injected mice treated with PLX5622 alleviates the motor deficits. These results are in line with previous studies that utilized another CSF1R inhibitor, PLX3397, and demonstrate that microglial depletion reduced exogenous αSyn in the substantia nigra^19,20^. Similarly, the microglial depletion by PLX3397 in 6-hydroxydopamine model showed improved sensory motor functions and depressive-like behavior^21^. Other studies have also shown that rosiglitazone, an anti-diabetic drug, inhibited the microglial release of interleukin 6 and reduced αSyn pathology, thereby preventing dopaminergic neuron loss^22^. Similarly, minocycline, an antimicrobial drug, decreased the proliferation and microglial activity, protected mice from dopaminergic neuron loss, increased the dopamine level, and decreased Lewy body pathology in mice^23^.

In addition, several studies have demonstrated the potential of CSF1R inhibitors in attenuating neurodegeneration in toxic PD models involving rotenone^24^, 6-hydroxydopamine^21^, and MPTP^25^. Given these references, there exists the possibility that the observed loss of TH neurons in the LPS-only group could be potentially reversible through the administration of CSF1R inhibitors. However, the precise impact of CSF1R inhibition on TH cell loss in the group exposed solely to LPS injection was not explored within the confines of the present study. It is important to note that conflicting results are present; a study has indicated that the use of CSF1R inhibitors might exacerbate disease outcomes^26^. Further investigation into the effects of CSF1R inhibitor treatment within the LPS-only treated group could yield valuable insight into whether microglial activity propels neurodegeneration, or if synucleinopathy plays a partial or intermediary role in the process of neurodegeneration.

Although an exhaustive downstream pathway remains to be unveiled, we hypothesize that the decrease in the internalization of fibrillar synuclein by microglia might curtail the generation of subsequent pathogenic forms of αSyn and their subsequent exocytosis. Nonetheless, further research is required to fully comprehend the mechanism underlying microglia linked to synucleinopathy in PD and to develop effective therapies.

To conclude, our study demonstrates that microglial reactivity hastens the aggregation and accumulation of αsyn, resulting in substantial neurodegeneration in the PD mouse model. Additionally, inhibition of microglial reactivity mitigates these alterations in the PFF-induced PD mouse model. Consequently, our findings suggest that microglia could be a therapeutic target for treating PD. However, more research is required to gain a better understanding of the detailed molecular mechanisms or subtype changes in microglia that contribute to αSyn pathology as well as the long-term effect of microglial inhibition in synucleinopathy and neurodegeneration.

## Methods

### Recombinant αSyn purification, fibril generation, and sonication

Recombinant full-length mouse αSyn monomer was purified using the same method as described in our previous study^27,28^. To generate PFF, the monomeric αSyn was shaken in an Eppendorf tube at 800 RPM for 10 days at 37 °C in the thermomixer inside an incubator. Just before injection, PFF was diluted in phosphate buffered saline (PBS) to a concentration of 2.5 µg/µl and sonicated at 37 °C for 10 min in a water bath (Grant XUBA3 Ultrasonic bath). The characterization of PFF was performed using Thioflavin T assay and transmission electron microscopy (TEM) imaging as previously described^27,28^.

### Animals

Male C57BL/6 mice, 10 weeks old, were obtained from Young Bio Company, South Korea, and used in the studies. The mice were housed in the animal facility of Hallym Medical Center, Hallym University, Korea, and were maintained at a constant room temperature (RT) with a 12-hour light-dark cycle. Fresh bedding and food were provided weekly until the time of sacrifice. All animal experiments were carried out in compliance with the animal research committee of Hallym University Sacred Heart Hospital (HMC2019-1-1130-42).

### Experimental design

Two sets of experiments were conducted to investigate the contribution of inflammation in the propagation of αSyn and neurodegeneration. In the first experiment, we aimed to induce additional inflammation by co-injecting LPS with PFF (*n* = 12 per time point), injecting PFF-only (*n* = 12 per time point), injecting LPS only (*n* = 12 per time point), or not injecting any substances (*n* = 12) into the right striatum. These mice were sacrificed at different time points (0, 14, 30, 90, 120, and 150 days post-injection, dpi) after injection (Fig. 1a).

In the second set of experiments, we investigated whether inhibition of microglia could reduce αSyn propagation and neurodegeneration. Mice were treated with oral gavage of PLX5622 (65 mg/kg) - an inhibitor of colony-stimulating factor 1 receptor (CSF1R), microglia inhibitor - for 2 weeks before and after PFF injection, totaling 4 weeks of treatment. This group was compared with mice treated with a control substance prepared with a mix of diluent (2% Hydroxypropyl Methylcellulose and 25% Polysorbate 80) and Dimethyl Sulfoxide (DMSO) instead of PLX5622.

### Stereotaxic injection of PFF and/or LPS into the striatum of mice

Lipopolysaccharide (LPS) from E. coli O127:B8 (cat# L3129 - 25 mg) was purchased from Sigma-Aldrich. LPS was first dissolved in distilled water at a concentration of 5 mg/ml, following the manufacturer’s instruction, and aliquots were stored at −20 °C. Mice were anesthetized with Avertin (Tribromoethanol, 240 mg/kg, 0.2 ml/10 g, i.p.) for unilateral injection of PFF and/or LPS into the striatum using a Hamilton microsyringe under stereotaxic surgery, as previously described^5,27,28^. Briefly, for PFF with LPS injection, just before injection, a solution containing 10 µg/2 µl of LPS mixed with 5 µg/2 µl of PFF was prepared, and 4 µl of the total solution was immediately delivered into the striatum at the following coordinates: −2 mm lateral, +0.5 mm posterior from the bregma, −3.6 mm below the dural surface, according to the mouse brain atlas. For PFF injection, PFF was diluted in PBS at a final concentration of 5 µg/4 µl, and a total 4 µl of the solution was immediately delivered into the striatum at the same coordinates as the PFF plus LPS inoculated mice. For LPS injection, LPS was diluted in PBS at a final concentration of 10 µg/4 µl, and 4 µl of the solution was immediately delivered into the striatum in the same coordinates as other injections.

In the second experiment, mice have inoculated with PFF (5 µg) or a control substance (PBS) into the right striatum, as described above.

### PLX5622 administration

To suppress microglial activity, the colony-stimulating factor-1 receptor (CSF1R) inhibitor, PLX5622 was obtained from MedChem Express and dissolved in DMSO at 130 mg/ml to create a stock solution. The drug was prepared according to the manufacturer’s protocol, as previously described in another study^29^, to create a working solution (6.5 mg/ml), the stock PLX5622 solution was diluted 20-fold in a diluent solution composed of 2% Hydroxypropyl Methylcellulose (09936-25 G, Sigma) and 25% Polysorbate 80 (P1754, Sigma) by adding 1 volume of stock solution (130 mg/ml) to 19 volumes of diluent. The working solution was then administered once per day via oral gavage at a dosage of 100 µl (6.5 mg/ml) per 10 grams of body weight, resulting in a final dosage of 65 mg/kg. A vehicle solution was prepared by mixing diluent and DMSO. The mice were divided into two experimental groups: the vehicle group and the PLX5622 group. PLX5622 or vehicle was administered by oral gavage for 2 weeks prior to PFF injection into the right striatum as described previously and continued until the day of sacrifice or 14 days post PFF injection.

### Behavioral assessment

Motor function changes from day 0 to 150 days post-injection in mice injected with PFF plus LPS, LPS only, and PFF only were assessed using the rotarod test, wire hang test, and hindlimb clasping test, which were conducted as previously described for motor performance assessment^27,28,30,31^. For time-course analysis of the behavioral phenotype, one cohort of animals was divided into 3 groups as follows: PFF plus LPS injection (*n* = 12), LPS injection (*n* = 12), and PFF injection (*n* = 12). A total of 36 mice in these three groups were tested before injection as the baseline. Subsequently, all groups underwent three rounds of behavior tests every month post-injection for five months (150 dpi).

In the second set of experiments, the baseline motor performance of the animals was assessed using the rotarod and wire hang test prior to PLX5622 treatment and at 14 days into the drug treatment. At 90 days post-injection, motor activities were evaluated again using the rotarod, wire hang test, and hindlimb clasping test.

### Tissue processing and histological evaluation

At each time point, mice were deeply anesthetized with Avertin solution (240 mg/kg, 0.2 ml/10 g, i.p.) and perfused transcardially with 50 mL of ice-cold 1 × PBS, followed by 30 mL of ice-cold 4% paraformaldehyde. Whole brains were then collected and fixed in 4% paraformaldehyde overnight at 4 °C. The brains were subsequently sliced into 40-µm sections using a vibratome, as previously described^27^, and stored in 0.05% sodium azide at 4 °C until use.

Immunohistochemistry was used to evaluate phosphorylated αSyn at Serine 129 (pSyn), ionized calcium-binding adaptor molecule1 (Iba1)-positive microglia, and tyrosine hydroxylase (TH)-positive dopaminergic nerve terminals on 40 µm sections, following the previously described protocol^27,28^. Sections were first blocked with peroxidase endogenous (3% ${{\rm{H}}}_{2}{{\rm{O}}}_{2})$ for 15 min at RT and then incubated with a blocking solution containing 2% BSA in 0.3% Triton-X/PBS for 1 h at RT. Next, primary antibodies were applied overnight at 4 °C, rabbit anti-pSyn S129 antibody (Abcam ab51253; 1: 1000), rabbit anti-Iba1 antibody (Wako, #016-20001; 1: 500), and mouse anti-TH antibody (Immunostar, #22941; 1: 1000). The brain sections were washed three times in PBS and were further incubated with suitable horseradish peroxidase- (HRP)- conjugated secondary antibodies for 2 hours at RT. After three PBS washes, staining was developed by a 3,3′-diaminobenzidine (DAB) reagent kit (Vector Laboratories), and sections were transferred onto coated slides. Finally, sections were covered with a non-aqueous mounting medium (DPX-Sigma, 100579) and stored at 4 °C.

Densitometric analysis of immunostaining in the striatum and amygdala were performed using ImageJ/Fiji, and the average optical density (OD) was calculated as previously described^27,28^. Briefly, color images were corrected by applying color deconvolution with the H-DAB vector function in ImageJ. The striatum or amygdala in four 40-µm sections from individual mice were measured by drawing the region of interest. The resulting gray values were converted to relative OD using the following formula OD = log (255/mean gray). Thus, the mean OD value was used as a measurement for microglial reactivity, pSyn density, and TH optical density. Additionally, the number of αSyn inclusions was counted in the substantia nigra section stained with pSyn.

Immunofluorescence was performed following the previously described protocol^27,28^. Tissue sections were incubated in blocking solution (1:1000 Normal donkey serum with 0.5% Triton-X-100 in PBS) for 1 h at RT, then incubated overnight at 4 °C with primary antibodies (pSyn S129, TH, and Iba1) in blocking solution. After three washing steps in PBS, sections were incubated with Alexa-conjugated secondary antibodies (AlexaFluor 555, AlexaFluor 488) for 2 h at RT. Sections were then washed three times and mounted on a microscope slide with a mounting medium containing 4′-6-diamidino-2-phenylindole (DAPI) for nuclei staining.

### Stereology analysis for TH neurons

Stereology analysis was used to assess the total number of TH -positive neurons in substantia nigra (SN), excluding the ventral tegmental area, every four sections (40 µm-thick) through the optical fractionator principle using Stereo Investigator Software 11 (MicroBrightField) as described previously^32^. The SN region was delineated in low magnification (2.5 × objective), and a point grid was overlaid onto each section. The SN region was acquired at 20× magnification, with a grid area of 150 × 150 µm and a counting frame size of 50 × 50 µm. The coefficient attributable to sampling was calculated according to Gundersen and Jensen (1987), and values less than 0.1 were accepted. A total of 5–6 animals per experimental group were included in this analysis.

### Western blot

Animals were euthanized using cervical dislocation and the head was separated. Brains were harvested and separated into injected (ipsilateral) and non-injected hemispheres (contralateral) excluding the cerebellum as previously described^27,28^. The brain was homogenized in the PBS buffer, and then it was dissolved in a non-ionic buffer (NP-40 soluble fraction) supplement with protease and phosphatase inhibitor cocktails as previously described. Samples were centrifuged at 12,000 RPM for 20 min at 4 °C. Supernatants used for soluble protein analysis and pellets were further homogenized in ionic buffers (NP-40 insoluble fraction). Samples were centrifuged at 12,000 RPM for 40 min at 4 °C and supernatants were collected for insoluble protein analysis.

For gel electrophoresis, protein samples were transferred onto a 0.2 µm PVDF membrane. Transferred blots were treated and incubated with primary antibodies as previously described^27,28^. Primary antibodies used for this study included rabbit anti-αSyn (Abcam ab212184; 1: 1000), rabbit anti-pSyn S129 (Abcam ab51253; 1: 1000), rabbit anti-Iba1 (Wako, #016-20001; 1: 1000), mouse anti-GFAP (Millipore, #MAB360; 1: 1000), and mouse anti-β-actin (Santa Cruz biotechnology, #sc47778; 1: 1000).

Membranes were then washed with Tris-buffered Saline with 0.1% Tween 20 detergent (TBS-T) and incubated with HRP-conjugated secondary antibodies for 1 h at RT. The membranes were washed again in TBS-T and the signal was visualized with ECL reagent using the Amersham Imager 600 Western Blot imaging system (GE Healthcare Life Sciences). All blots were processed in parallel and derived from the same experiments. Analysis of blot was performed in Fiji/ImageJ. The protein of interest signal was normalized to its respective loading control (beta-actin). Original blots are presented in Supplementary Fig. 8.

### Statistics

Statistical analysis and graphing were performed using GraphPad Prism software version 9.4.0. All data presented in the graphs are shown as means ± SEM. Detailed statistical analyses are provided in Supplementary Table 1. Statistical differences in means were assessed using the test specified in the figure legends, with significance levels indicated as follows: # for *p* < 0.05, ## for *p* < 0.01, ### for *p* < 0.001, #### for *p* < 0.0001, when compared to mice without any injection (no injection) or baseline. For comparison among the three different animal models, significance levels were noted as * for *p* < 0.05, ** for *p* < 0.01, *** for *p* < 0.001, and **** for *p* < 0.0001. Non-significant results were denoted as ‘ns’.

### Reporting summary

Further information on research design is available in the Nature Research Reporting Summary linked to this article.

### Supplementary information

Supplemental Data

Reporting summary

## Data Availability

All data generated or analyzed during this study are included in this published article and its Supplementary Information files.
