# Supplementary material for: Microglial inhibition alleviates alpha-synuclein propagation and neurodegeneration in Parkinson’s disease mouse model
Source: NPJ Parkinsons Dis. 2024 Feb 2;10:32. doi: 10.1038/s41531-024-00640-2 (PMC10834509; doi:10.1038/s41531-024-00640-2)

Microglial inhibition alleviates alpha-synuclein propagation and neurodegeneration in Parkinson's Disease mouse model

Supplementary Table 1: Main results of statistical analysis

Figure	Test results		Multiple comparison results		
Fig. 1c	One-way ANOVA	F *(3, 20)* = 135<br>*p* <0.0001	Tukey *post hoc*	LPS vs no injection	*p* < 0.0001
				PFF+LPS vs no injection	*p* < 0.0001
				PFF vs no injection	*p* < 0.0001
				LPS vs PFF+LPS	*p* < 0.0001
				LPS vs PFF	*p* = 0.06
				PFF vs PFF+LPS	*p* < 0.0001
Fig. 1d	One-way ANOVA	F *(3, 20)* = 19.45<br>*p* < 0.0001	Tukey *post hoc*	LPS vs no injection	*p* = 0.003
				PFF+LPS vs no injection	*p* < 0.0001
				PFF vs no injection	*p* = 0.0146
				LPS vs PFF+LPS	*p* = 0.0107
				LPS vs PFF	*p* = 0.8925
				PFF vs PFF+LPS	*p* = 0.0022
Fig. 1f	One-way ANOVA	F *(3, 20)* = 65.06<br>*p* < 0.0001	Šídák's *post hoc*	LPS vs no injection	*p* < 0.0001
				PFF+LPS vs no injection	*p* < 0.0001
				PFF vs no injection	*p* < 0.0001
				LPS vs PFF+LPS	*p* = 0.9979
				LPS vs PFF	*p* > 0.999
				PFF vs PFF+LPS	*p* > 0.999
Fig. 1g	One-way ANOVA	F *(3, 20)* = 14.48<br>*p* < 0.0001	Šídák's *post hoc*	LPS vs no injection	*p* = 0.0139
				PFF+LPS vs no injection	*p* =0.0007
				PFF vs no injection	*p* = 0.9995
				LPS vs PFF+LPS	*p* = 0.6461
				LPS vs PFF	*p* = 0.0065

				PFF vs PFF+LPS	$p = 0.0003$
Fig. 2a (14dpi)	One-way ANOVA	F *(3, 20)* = 175.5 $p < 0.0001$	Tukey *post hoc*	LPS vs no injection	$p = 0.34$
				PFF+LPS vs no injection	$p < 0.0001$
				PFF vs no injection	$p < 0.0001$
				LPS vs PFF+LPS	$p < 0.0001$
				LPS vs PFF	$p < 0.0001$
				PFF vs PFF+LPS	$p = 0.0208$
Fig. 2a (90dpi)	One-way ANOVA	F *(3, 20)* = 146.2 $p < 0.0001$	Tukey *post hoc*	LPS vs no injection	$p = 0.1509$
				PFF+LPS vs no injection	$p < 0.0001$
				PFF vs no injection	$p < 0.0001$
				LPS vs PFF+LPS	$p < 0.0001$
				LPS vs PFF	$p < 0.0001$
				PFF vs PFF+LPS	$p = 0.0273$
Fig. 2a (150dpi)	One-way ANOVA	F *(3, 20)* = 62.95 $p < 0.0001$	Tukey *post hoc*	LPS vs no injection	$p = 0.1984$
				PFF+LPS vs no injection	$p < 0.0001$
				PFF vs no injection	$p < 0.0001$
				LPS vs PFF+LPS	$p < 0.0001$
				LPS vs PFF	$p = 0.0007$
				PFF vs PFF+LPS	$p < 0.0001$
Fig. 2c (14dpi)	One-way ANOVA	F *(3, 20)* = 37.03 $p < 0.0001$	Tukey *post hoc*	LPS vs no injection	$p > 0.9999$
				PFF+LPS vs no injection	$p < 0.0001$
				PFF vs no injection	$p < 0.0001$
				LPS vs PFF+LPS	$p < 0.0001$
				LPS vs PFF	$p < 0.0001$
				PFF vs PFF+LPS	$p = 0.0382$
Fig. 2c (90dpi)	One-way ANOVA	F *(3, 20)* = 102.5 $p < 0.0001$	Tukey *post hoc*	LPS vs no injection	$p > 0.9999$
				PFF+LPS vs no injection	$p < 0.0001$
				PFF vs no injection	$p < 0.0001$
				LPS vs PFF+LPS	$p < 0.0001$
				LPS vs PFF	$p < 0.0001$
				PFF vs PFF+LPS	$p = 0.044$
Fig. 2c (150dpi)	One-way ANOVA	F *(3, 20)* = 138.2 $p < 0.0001$	Tukey *post hoc*	LPS vs no injection	$p = 0.8261$
				PFF+LPS vs no injection	$p < 0.0001$
				PFF vs no injection	$p < 0.0001$

				LPS vs PFF+LPS	*p* < 0.0001
				LPS vs PFF	*p* < 0.0001
				PFF vs PFF+LPS	*p* = 0.9995
Fig. 3b	One-way ANOVA	F *(3, 20)* = 80.98<br>*p* < 0.0001	Šídák's *post hoc*	LPS vs no injection	*p* = 0.0001
				PFF+LPS vs no injection	*p* < 0.0001
				PFF vs no injection	*p* < 0.0001
				LPS vs PFF+LPS	*p* = 0.0242
				LPS vs PFF	*p* = 0.521
				PFF vs PFF+LPS	*p* = 0.0414
Fig. 3c	One-way ANOVA	F *(3, 20)* = 18.89<br>*p* < 0.0001	Šídák's *post hoc*	LPS vs no injection	*p* < 0.0001
				PFF+LPS vs no injection	*p* < 0.0001
				PFF vs no injection	*p* < 0.0001
				LPS vs PFF+LPS	*p* = 0.9830
				LPS vs PFF	*p* = 0.9681
				PFF vs PFF+LPS	*p* > 0.9999
Fig. 3d	Two-way repeated ANOVA	Main effect of days post injection<br><br>F(*3.800, 125.4*) = 22.95<br>*p* < 0.0001	Tukey *post hoc*	LPS-injected mice at 150 dpi vs 0 dpi	*p* = 0.0007
				PFF+LPS-injected mice at 30 dpi vs 0 dpi	*p* = 0.0006
				PFF+LPS-injected mice at 60, 90, 120 dpi vs 0 dpi	*p* < 0.0001
				PFF+LPS-injected mice at 150 dpi vs 0 dpi	*p* = 0.0001
				PFF-injected mice at 120 dpi vs 0 dpi	*p* = 0.0153
				PFF-injected mice at 150 dpi vs 0 dpi	*p* = 0.0036
		Main effect of the injection (three different models)<br>F(*2, 33*) = 1.924<br>*p* = 0.1621		PFF+LPS-injected mice vs LPS-injected mice at 60 dpi	*p* = 0.036
Fig. 3e	two-way repeated ANOVA	Main effect of the injection<br><br>F(2, 33) = 0.2555<br>*p* = 0.7760	Tukey *post hoc*		
		Main effect of days post injection	Tukey *post hoc*	LPS-injected mice at 30 dpi vs 120 dpi	*p* = 0.0065

		F (3.692, 121.9) = 21.75<br>*p* < 0.0001		PFF+LPS-injected mice at 90 dpi vs 0 dpi	*p* = 0.0123
				PFF+LPS-injected mice at 120 dpi vs 0 dpi	*p* = 0.0302
				PFF+LPS-injected mice at 150 dpi vs 0 dpi	*p* = 0.0262
				PFF+LPS-injected mice at 30 dpi vs 90 dpi	*p* = 0.0159
				PFF+LPS-injected mice at 30 dpi vs 120 dpi	*p* = 0.0444
				PFF+LPS-injected mice at 30 dpi vs 150 dpi	*p* = 0.0166
				PFF-injected mice at 120 dpi vs 0 dpi	*p* = 0.0261
				PFF-injected mice at 150 dpi vs 0 dpi	*p* = 0.0187
				PFF-injected mice at 30 dpi vs 90 dpi	*p* = 0.0013
				PFF-injected mice at 30 dpi vs 120 dpi	*p* = 0.0016
				PFF-injected mice at 30 dpi vs 150 dpi	*p* = 0.0024
Fig. 3f	Two-way repeated ANOVA	Main effect of days post injection<br><br>F(2*.961, 97.72*) = 94.74<br>*p* < 0.0001	Tukey *post hoc*	LPS-injected mice at 30 dpi vs 0 dpi	*p* = 0.0265
				LPS-injected mice at 60 dpi vs 0 dpi	*p* = 0.0025
				LPS-injected mice at 90 dpi vs 0 dpi	*p* = 0.0047
				LPS-injected mice at 120 dpi vs 0 dpi	*p* = 0.0004
				LPS-injected mice at 150 dpi vs 0 dpi	*p* = 0.0007
				PFF+LPS-injected mice at 30 dpi vs 0 dpi	*p* = 0.0084
				PFF+LPS-injected mice at 60 dpi vs 0 dpi	*p* = 0.0024
				PFF+LPS-injected mice at 90 dpi vs 0 dpi	*p* = 0.0002
				PFF+LPS-injected mice at 120 dpi vs 0 dpi	*p* = 0.0002
				PFF+LPS-injected mice at 150 dpi vs 0 dpi	*p* = 0.0001
				PFF-injected mice at 30 dpi vs 0 dpi	*p* = 0.0712

				PFF-injected mice at 60 dpi vs 0 dpi	*p* = 0.0057
				PFF-injected mice at 90 dpi vs 0 dpi	*p* = 0.0008
				PFF-injected mice at 120 dpi vs 0 dpi	*p* < 0.0001
				PFF-injected mice at 150 dpi vs 0 dpi	*p* < 0.0001
		Main effect of the injection<br><br>F(2, 33) = 15.23<br>*p* < 0.0001	Tukey *post hoc*	PFF+LPS-injected mice vs PFF-injected mice at 60 dpi	*p* = 0.0095
				PFF+LPS-injected mice vs LPS-injected mice at 90 dpi	*p* = 0.0066
				PFF+LPS-injected mice vs PFF-injected mice at 90 dpi	*p* = 0.0101
				PFF+LPS-injected mice vs LPS-injected mice at 120 dpi	*p* = 0.0053
				PFF+LPS-injected mice vs PFF-injected mice at 120 dpi	*p* = 0.0087
				PFF+LPS-injected mice vs LPS-injected mice at 150 dpi	*p* = 0.005
				PFF+LPS-injected mice vs PFF-injected mice at 150 dpi	*p* = 0.5985
				LPS-injected mice vs PFF-injected mice at 150 dpi	*p* = 0.0126
Fig. 4c	Mann-Whitney	*p* = 0.0043			
Fig. 4d	One-way ANOVA	F (3, 143) = 0.1891<br>*p* = 0.9037	Tukey *post hoc*	Pre-treated PLX vs 14 days treated PLX	*p* = 0.9707
				Pre-treated vehicle vs 14 days treated vehicle	*p* = 0.9386
				14 days-treated PLX5622 vs. 14 days-treated vehicle	*p* = 0.9112
Fig. 4e	One-way ANOVA	F (3, 143) = 1.428<br>*p* = 0.2371	Tukey *post hoc*	Pre-treated PLX vs 14 days treated PLX	*p* = 0.9336
				Pre-treated vehicle vs 14 days treated vehicle	*p* = 0.9285

				14 days-treated PLX5622 vs. 14 days-treated vehicle	*p* = 0.2286
Fig. 5d (ipsilateral)	Mann Whitney	*p* = 0.0294			
Fig. 5d (contralateral)	Mann Whitney	*p* = 0.6623			
Fig. 5f (ipsilateral)	Mann Whitney	*p* = 0.0303			
Fig. 5f (contralateral)	Mann Whitney	*p* = 0.0087			
Fig. 5h (ipsilateral)	Mann Whitney	*p* = 0.0173			
Fig. 5j (ipsilateral)	One-way ANOVA	F (2, 14) = 27.86 *p* < 0.0001	Tukey *post hoc*	PFF+PLX vs no injection	*p* = 0.0032
				PFF+Veh vs no injection	*p < 0.0001*
				PFF+PLX vs PFF+Veh	*p* = 0.0224
Fig. 5j (contralateral)	One-way ANOVA	F (2, 14) = 1.141 *p* = 0.3476	Tukey *post hoc*	PFF+PLX vs no injection	*p* = 0.5571
				PFF+Veh vs no injection	*p* = 0.3418
				PFF+PLX vs PFF+Veh	*p* = 0.9410
Fig. 5l (ipsilateral)	One-way ANOVA	F (2, 14) = 9.203 *p* = 0.0028	Tukey *post hoc*	PFF+PLX vs no injection	*p* = 0.2813
				PFF+Veh vs no injection	*p* = 0.0021
				PFF+PLX vs PFF+Veh	*p* = 0.0654

Fig. 5l (contralateral)	One-way ANOVA	F (2, 14) = 2.581<br>*p* = 0.1111	Tukey *post hoc*	PFF+PLX vs no injection	*p* = 0.8013
				PFF+Veh vs no injection	*p* = 0.1020
				PFF+PLX vs PFF+Veh	*p* = 0.3303
Fig. 5m (rota rod)	One-way ANOVA	F (3, 18) = 2.512<br>*p* = 0.0912	Tukey *post hoc*	PFF+Veh vs pre-surgery	*p* = 0.1849
				PFF+PLX vs pre-surgery	*p* = 0.3883
				PFF+Veh vs PFF+PLX	*p* = 0.8687
Fig. 5m (wire hang)	One-way ANOVA	F (3, 18) = 4.772<br>*p* = 0.0128	Tukey *post hoc*	PFF+Veh vs pre-surgery	*p* = 0.0270
				PFF+PLX vs pre-surgery	*p* = 0.2160
				PFF+Veh vs PFF+PLX	*p* = 0.8656
Fig. 5m (clasping test)	One-way ANOVA	F (3, 18) = 19.57<br>*p* < 0.0001	Tukey *post hoc*	PFF+Veh vs pre-surgery	*p* < 0.0001
				PFF+PLX vs pre-surgery	*p* = 0.0632
				PFF+Veh vs PFF+PLX	*p* = 0.0107

Microglial inhibition alleviates alpha-synuclein propagation and neurodegeneration in Parkinson's Disease mouse model

Supplementary Fig. 1: Western blot analysis of GFAP-positive astrocyte in the whole brain homogenate at 14 dpi. Statistical analyses were performed by using one-way ANOVA together with a post-hoc-test for multiple comparisons, whereas (#) denotes a significant difference compared with no injection, and (*) denotes a significant difference between three animal models.

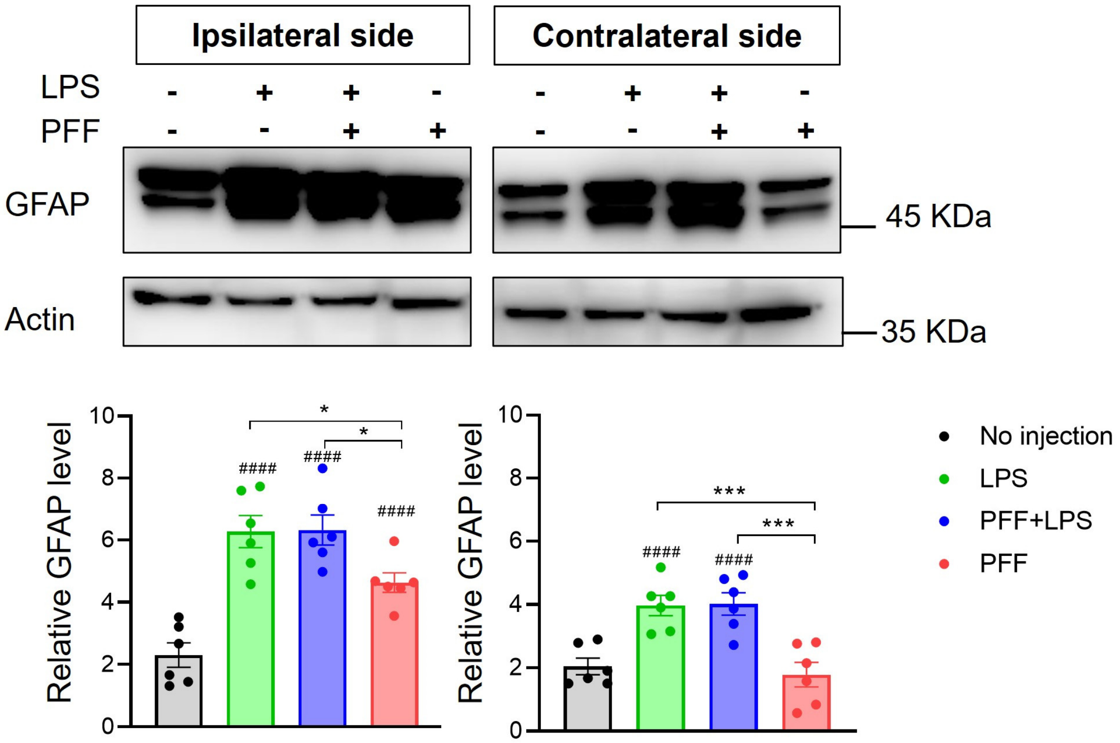

Supplementary Fig. 2: Representative image for pSyn (S129) in the striatum and substantia nigra in LPS-only, PFF with LPS, and PFF-only injected mice at different time points after injection. The white arrow signifies the ipsilateral side where LPS or PFF injection was administered. Scale bar: 1 mm.

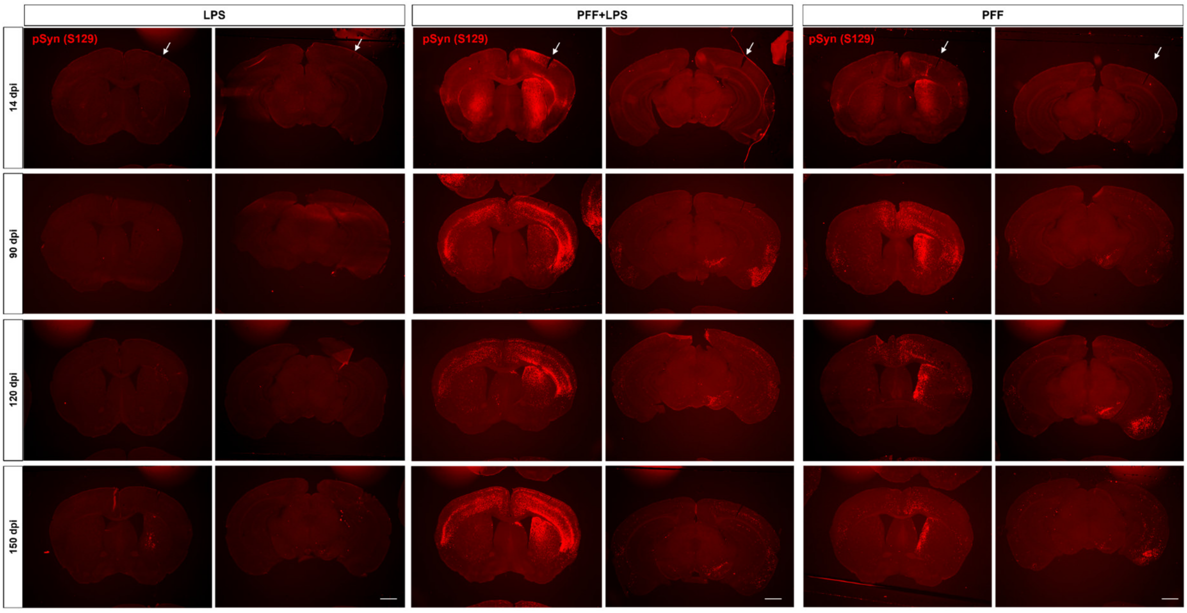

Supplementary Fig. 3: Representative image and its quantification for pSyn (S129) in the striatum and substantia nigra in LPS-only, PFF with LPS, and PFF-only injected mice at 30 and 120 dpi and no injection. Scale bar: 200 µm.

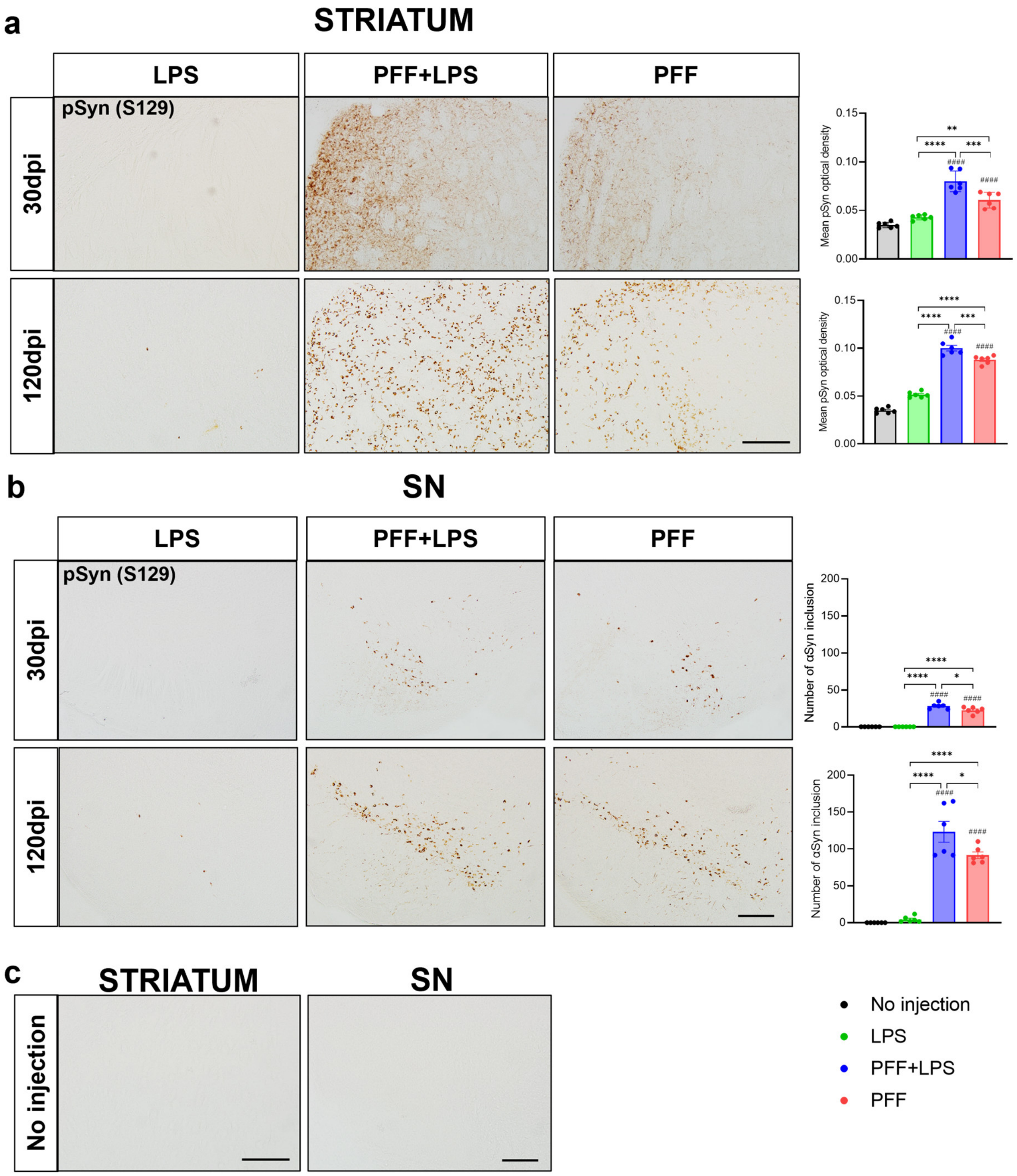

Supplementary Fig. 4: **a** Presence of pSyn (S129) immunoreactive aggregates in the ipsilateral striatum of LPS-injected mice at 120 dpi. Scale bar: 1 mm, 200 µm. **b** Representative image for pSyn (S129) and TH immunoreactivity in SN at 120 dpi. The white arrow signifies the ipsilateral side where LPS injection was administered. Scale bar: 200 µm. **c, d** Stereology count of TH-positive cells in ipsilateral (**c**) and contralateral (**d**) SN compacta. Statistical analyses were performed using one-way ANOVA followed by a post-hoc-test for multiple comparisons. (*) denotes a significant difference between three different animal models. (#) denotes a significant difference compared to the mice without injection.

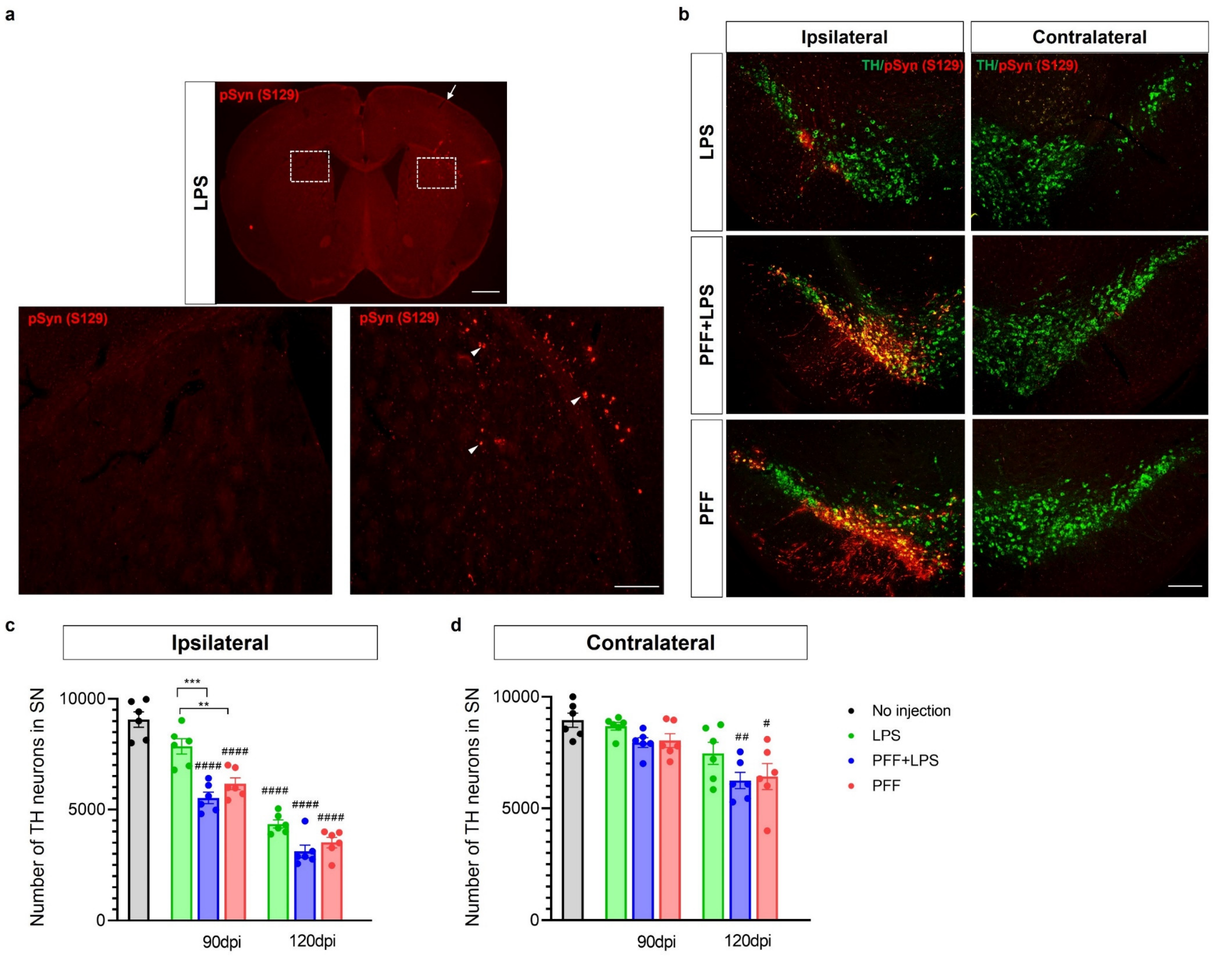

Supplementary Fig. 5: Extended administration of PLX5622 until 7 and 14 dpi after PFF or PBS injection partially depleted microglia. (**a**) Overview coronal sections showing Iba1-positive microglia in PFF or PBS injection mice. The white arrow signifies the ipsilateral side where PBS or PFF injection was administered. (**b, c**) Higher magnification images showing the loss of microglial in PLX5622 treated mice. Scale bar: 1 mm, 200 μm.

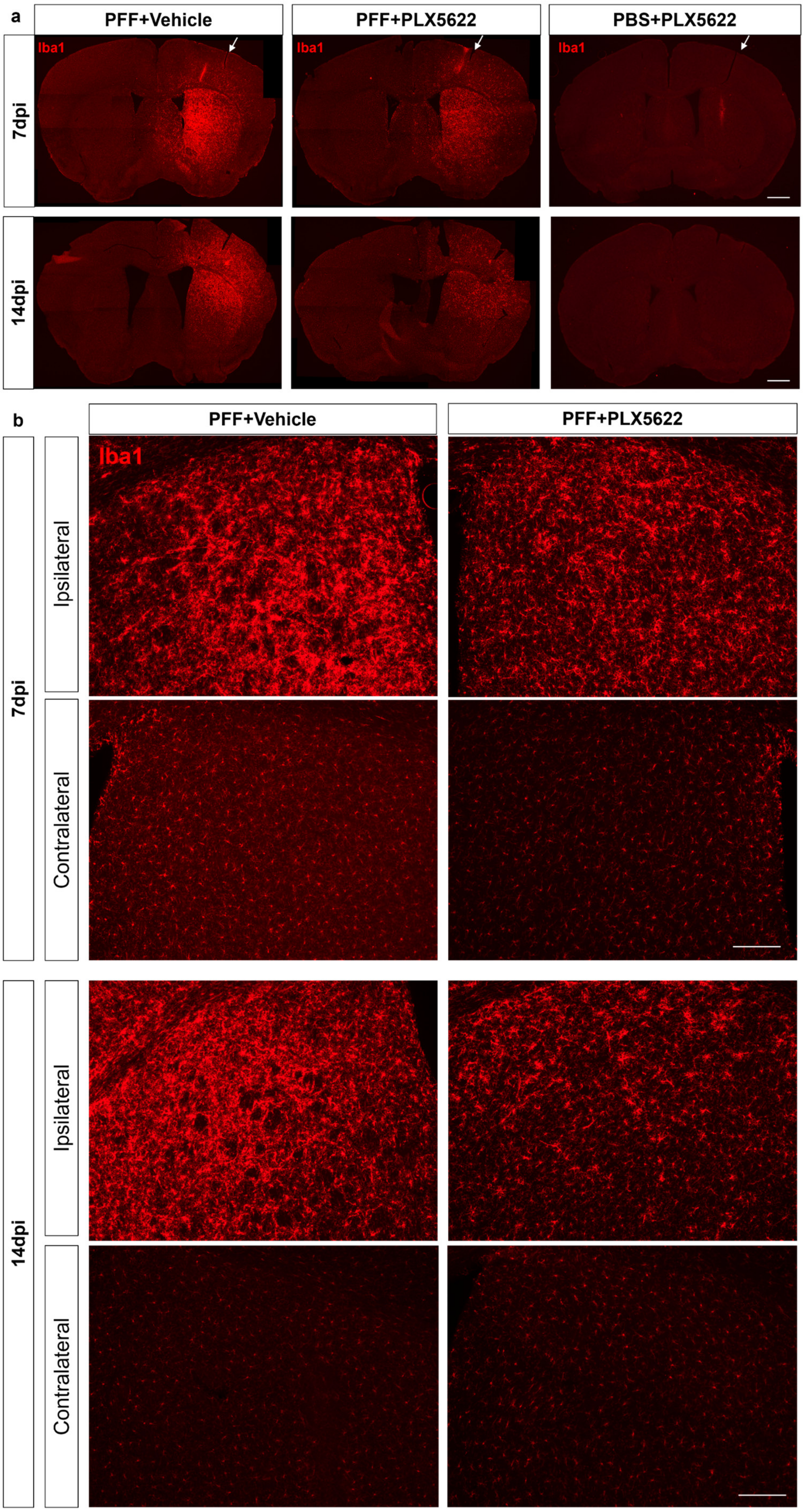
a
PFF+Vehicle
PFF+PLX5622
PBS+PLX5622
Iba1
7dpi
14dpi
b
PFF+Vehicle
PFF+PLX5622
Iba1
7dpi
Ipsilateral
Contralateral
14dpi
Ipsilateral
Contralateral

Supplementary Fig. 6: Representative images and quantification of microglial reaction in the striatum, amygdala, and substantia nigra after 90 days of PFF injection for both PLX5622 and vehicle treatment (n= 2 or 3). Statistics were not included. Scale bar: 200 μm.

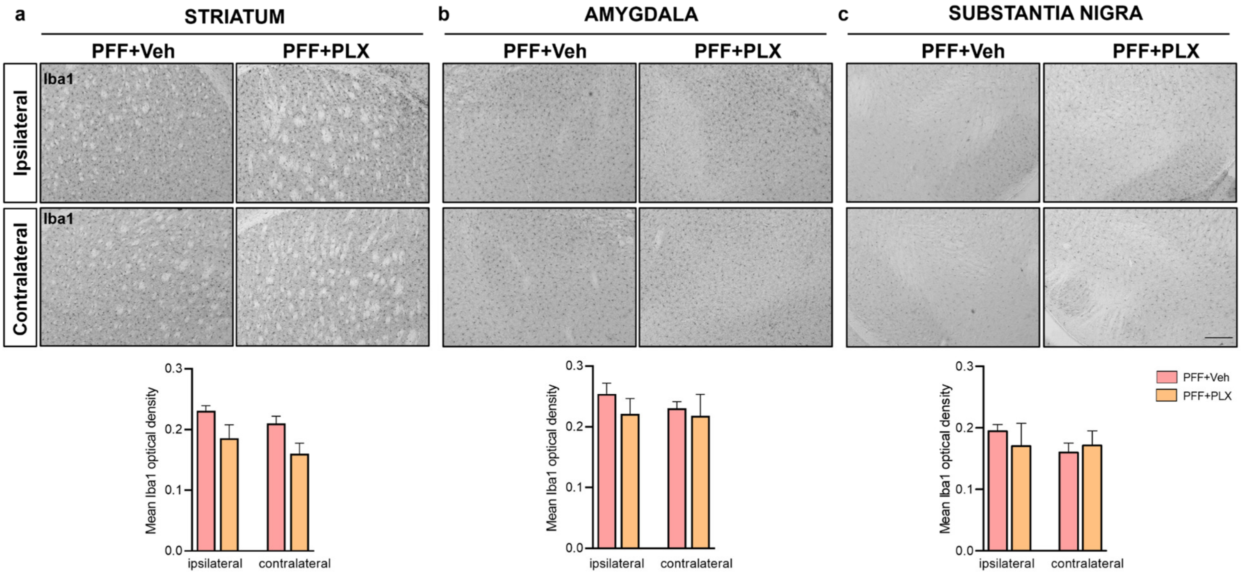

Supplementary Fig. 7: PLX5622 administration in mice delayed αSyn propagation. **a** Representative fluorescence images of pSyn in the brain at 7 dpi and 14 dpi. Scale bar: 200 μm. **b-e** Western blot analysis and quantification of αSyn level in NP-40 soluble and NP-40 insoluble fractions at 14dpi. Statistical analysis was performed by one-way ANOVA followed by multiple comparison post hoc test, whereas (#) denotes a significant difference compared to no injection, and (*) denotes a significant difference between treatments.

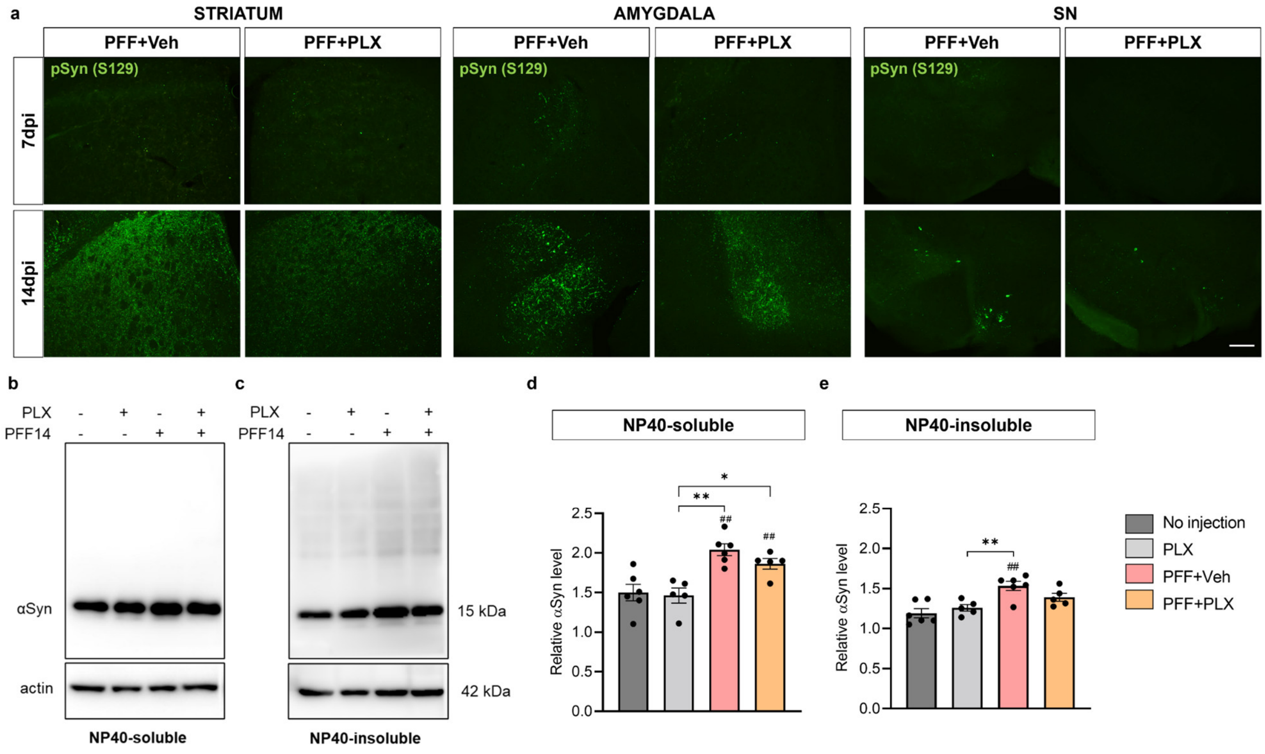
a
STRIATUM
AMYGDALA
SN
PFF+Veh
PFF+PLX
pSyn (S129)
7dpi
14dpi
b
c
PLX
PFF14
αSyn
actin
15 kDa
42 kDa
NP40-soluble
NP40-insoluble
d
e
Relative αSyn level
No injection
PLX
PFF+Veh
PFF+PLX

Supplementary Fig. 8: Original full blots of western blot.

Fig.1e and Supplementary Fig.1

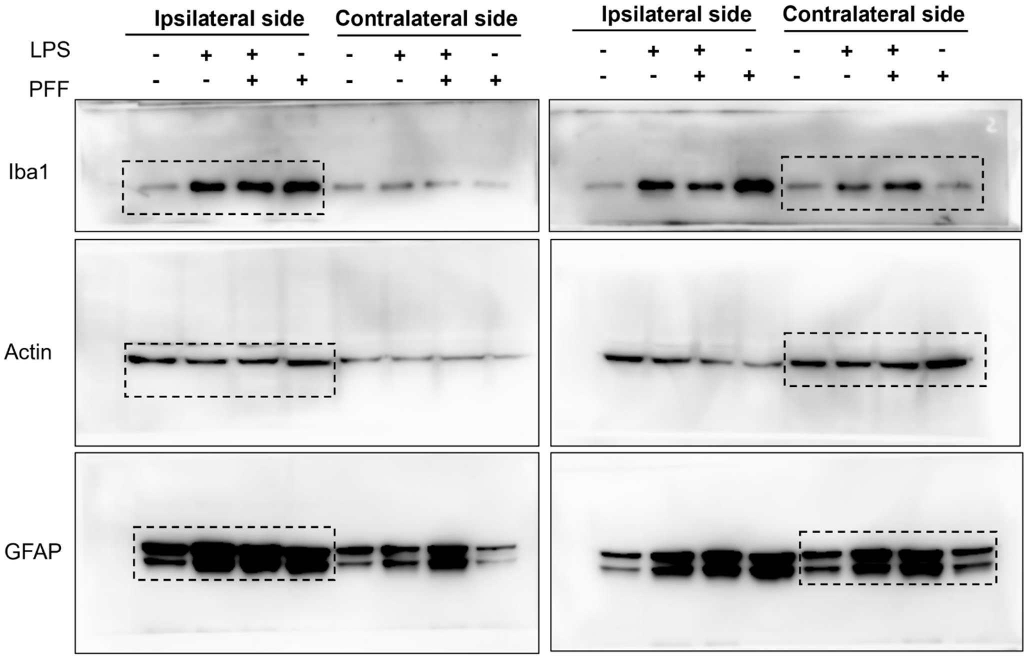

Fig. 4c

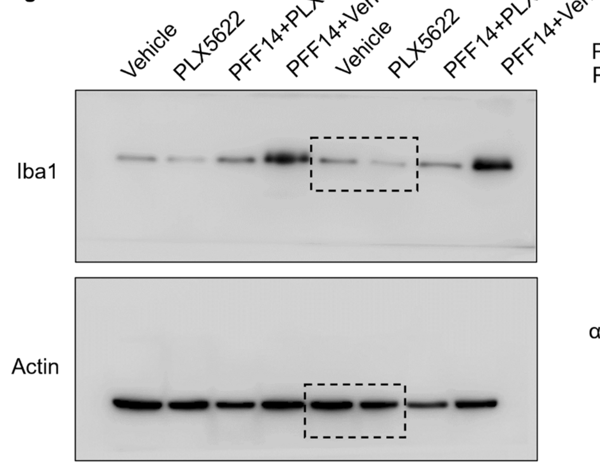

Supplementary Fig.7b and c

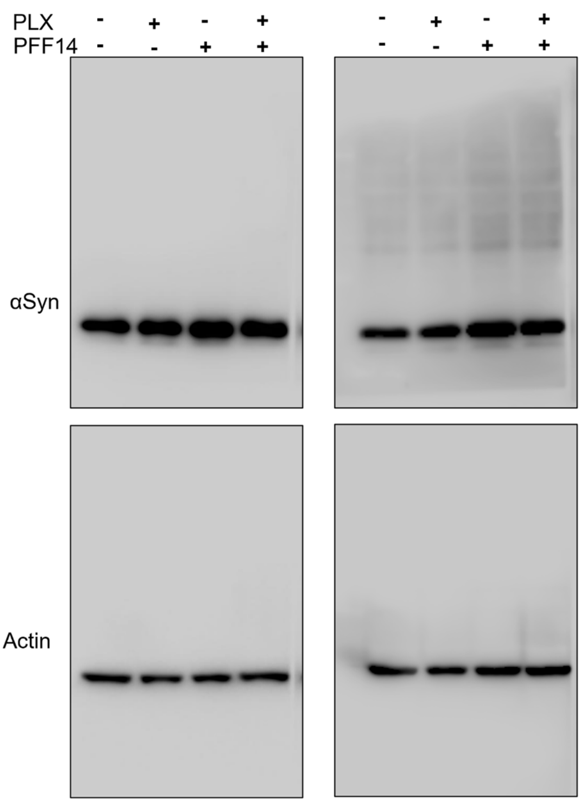

Supplement: Supplementary file 1 — Supplemental Data [file 41531_2024_640_MOESM1_ESM.pdf]
