# Supplementary material for: Microglial inhibition alleviates alpha-synuclein propagation and neurodegeneration in Parkinson’s disease mouse model
Source: NPJ Parkinsons Dis. 2024 Feb 2;10:32. doi: 10.1038/s41531-024-00640-2 (PMC10834509; doi:10.1038/s41531-024-00640-2)
Supplement: Supplementary file 2 — Reporting summary [file 41531_2024_640_MOESM2_ESM.pdf]

Corresponding author(s): Young Eun Kim

Last updated by author(s): Jan 10, 2024

Reporting Summary

Nature Portfolio wishes to improve the reproducibility of the work that we publish. This form provides structure for consistency and transparency in reporting. For further information on Nature Portfolio policies, see our Editorial Policies and the Editorial Policy Checklist.

Statistics

For all statistical analyses, confirm that the following items are present in the figure legend, table legend, main text, or Methods section.

n/a	Confirmed	
☐	☒	The exact sample size (*n*) for each experimental group/condition, given as a discrete number and unit of measurement
☐	☒	A statement on whether measurements were taken from distinct samples or whether the same sample was measured repeatedly
☐	☒	The statistical test(s) used AND whether they are one- or two-sided<br>*Only common tests should be described solely by name; describe more complex techniques in the Methods section.*
☐	☒	A description of all covariates tested
☐	☒	A description of any assumptions or corrections, such as tests of normality and adjustment for multiple comparisons
☐	☒	A full description of the statistical parameters including central tendency (e.g. means) or other basic estimates (e.g. regression coefficient) AND variation (e.g. standard deviation) or associated estimates of uncertainty (e.g. confidence intervals)
☐	☒	For null hypothesis testing, the test statistic (e.g. *F*, *t*, *r*) with confidence intervals, effect sizes, degrees of freedom and *P* value noted<br>*Give P values as exact values whenever suitable.*
☒	☐	For Bayesian analysis, information on the choice of priors and Markov chain Monte Carlo settings
☒	☐	For hierarchical and complex designs, identification of the appropriate level for tests and full reporting of outcomes
☒	☐	Estimates of effect sizes (e.g. Cohen's *d*, Pearson's *r*), indicating how they were calculated

Our web collection on statistics for biologists contains articles on many of the points above.

Software and code

Policy information about availability of computer code

Data collection	No software was used for data collection.
Data analysis	GraphPad Prism software version 9.4.0.

For manuscripts utilizing custom algorithms or software that are central to the research but not yet described in published literature, software must be made available to editors and reviewers. We strongly encourage code deposition in a community repository (e.g. GitHub). See the Nature Portfolio guidelines for submitting code & software for further information.

Data

Policy information about availability of data

All manuscripts must include a data availability statement. This statement should provide the following information, where applicable:

- Accession codes, unique identifiers, or web links for publicly available datasets
- A description of any restrictions on data availability
- For clinical datasets or third party data, please ensure that the statement adheres to our policy

The data used in this study are available on request from the corresponding authors.

Research involving human participants, their data, or biological material

Policy information about studies with human participants or human data. See also policy information about sex, gender (identity/presentation), and sexual orientation and race, ethnicity and racism.

Reporting on sex and gender	This study did not include human data.
Reporting on race, ethnicity, or other socially relevant groupings	*Please specify the socially constructed or socially relevant categorization variable(s) used in your manuscript and explain why they were used. Please note that such variables should not be used as proxies for other socially constructed/relevant variables (for example, race or ethnicity should not be used as a proxy for socioeconomic status).*<br>*Provide clear definitions of the relevant terms used, how they were provided (by the participants/respondents, the researchers, or third parties), and the method(s) used to classify people into the different categories (e.g. self-report, census or administrative data, social media data, etc.)*<br>*Please provide details about how you controlled for confounding variables in your analyses.*
Population characteristics	*Describe the covariate-relevant population characteristics of the human research participants (e.g. age, genotypic information, past and current diagnosis and treatment categories). If you filled out the behavioural & social sciences study design questions and have nothing to add here, write "See above."*
Recruitment	*Describe how participants were recruited. Outline any potential self-selection bias or other biases that may be present and how these are likely to impact results.*
Ethics oversight	*Identify the organization(s) that approved the study protocol.*

Note that full information on the approval of the study protocol must also be provided in the manuscript.

Field-specific reporting

Please select the one below that is the best fit for your research. If you are not sure, read the appropriate sections before making your selection.

☒ Life sciences ☐ Behavioural & social sciences ☐ Ecological, evolutionary & environmental sciences

For a reference copy of the document with all sections, see nature.com/documents/nr-reporting-summary-flat.pdf

Life sciences study design

All studies must disclose on these points even when the disclosure is negative.

Sample size	Based on the difference of our previous literatures and considering 95% CI and 5% accuracy, for the comparison of two groups' mean, we calculated sample size on this experiment.
Data exclusions	No data were excluded in this study.
Replication	Each experiment presented in the paper was repeated in multiple animals (between 5 to 6).<br>To ensure the reliability of our data, various brain sections from each mouse were stained for tissue analysis. In particular, four 40-µm slices from each independent mouse was measured for IHC analysis.<br>Each mouse underwent three trials for Western blot experiments, and each experiment was replicated in multiple mice.
Randomization	We allocated mice into different groups randomly. And all the surgery, and treatment were conducted at the same time in all groups.
Blinding	Investigation were not blinded to investigator during data collection and analysis.

Reporting for specific materials, systems and methods

We require information from authors about some types of materials, experimental systems and methods used in many studies. Here, indicate whether each material, system or method listed is relevant to your study. If you are not sure if a list item applies to your research, read the appropriate section before selecting a response.

Materials & experimental systems

n/a	Involved in the study	
☐	☒	Antibodies
☒	☐	Eukaryotic cell lines
☒	☐	Palaeontology and archaeology
☐	☒	Animals and other organisms
☒	☐	Clinical data
☒	☐	Dual use research of concern
☒	☐	Plants

Methods

n/a	Involved in the study	
☒	☐	ChIP-seq
☒	☐	Flow cytometry
☒	☐	MRI-based neuroimaging

Antibodies

Antibodies used

All anitbodies used has been included in the method section of this manuscript.
rabbit anti-pSyn S129 antibody (Abcam ab51253; 1:1000), rabbit anti-Iba1 antibody (Wako, #016-20001; 1:500), and mouse anti-TH antibody (Immunostar, #22941; 1:1000). rabbit anti-αSyn (Abcam ab212184; 1:1000), mouse anti-GFAP (Millipore, #MAB360; 1: 1000), and mouse anti-β-actin (Santa Cruz biotechnology, #sc47778; 1:1000).

Validation

1, abbit anti-pSyn S129 antibody (Abcam ab51253; 1:1000): https://www.abcam.com/alpha-synuclein-phospho-s129-antibody-ep1536y-ab51253.html
2, rabbit anti-Iba1 antibody (Wako, #016-20001; 1:500): https://labchem-wako.fujifilm.com/europe/product/detail/W01W0101-2000.html
3, mouse anti-TH antibody (Immunostar, #22941; 1:1000): https://www.immunostar.com/product/tyrosine-hydroxylase-antibody/
4, rabbit anti-αSyn (Abcam ab212184; 1:1000): https://www.abcam.com/products/primary-antibodies/alpha-synuclein-antibody-epr20535-ab212184.html
5,mouse anti-GFAP (Millipore, #MAB360; 1:1000): https://www.merckmillipore.com/DE/de/product/Anti-Glial-Fibrillary-Acidic-Protein-Antibody-clone-GA5,MM_NF-MAB360
6, mouse anti-β-actin (Santa Cruz biotechnology, #sc47778; 1:1000); https://www.scbt.com/p/beta-actin-antibody-c4

Animals and other research organisms

Policy information about studies involving animals; ARRIVE guidelines recommended for reporting animal research, and Sex and Gender in Research

Laboratory animals

mice, C57BL/6 , 10 weeks old

Wild animals

This study did not involve wild animals.

Reporting on sex

This study exclusively utilized male mice to eliminate potential sex-related variations and minimize confounding factors. The total number of mice employed for this study was 238.

Field-collected samples

This study did not involved samples from the field.

Ethics oversight

All animal experiments were carried out in compliance with the animal research committee of Hallym University Sacred Heart Hospital (HMC2019-1-1130-42).

Note that full information on the approval of the study protocol must also be provided in the manuscript.
